# The Effect of Physicochemical Modification on the Function of Antibodies Induced by Anti-Nicotine Vaccine in Mice

**DOI:** 10.3390/vaccines5020011

**Published:** 2017-05-17

**Authors:** Jennifer M. Thorn, Keshab Bhattacharya, Renata Crutcher, Justin Sperry, Colleen Isele, Barbara Kelly, Libbey Yates, James Zobel, Ningli Zhang, Heather L. Davis, Michael J. McCluskie

**Affiliations:** 1 Pfizer Biotherapeutics Pharmaceutical Sciences, Chesterfield, MO 63017, USA; keshab.bhattacharya@pfizer.com (K.B.); renata.i.williams@pfizer.com (R.C.); justin.sperry@pfizer.com (J.S.); colleen.isele@pfizer.com (C.I.); barbara.a.kelly@pfizer.com (B.K.); libbey.a.yates@pfizer.com (L.Y.); james.f.zobel@pfizer.com (J.Z.); 2 Pfizer Vaccine Immunotherapeutics, Ottawa Laboratories, Ottawa, ON K2K 3A2, Canada; nl_zhang@yahoo.com (N.Z.); Heather.Davis@Seqirus.com (H.L.D.); Michael.McCluskie@nrc-cnrc.gc.ca (M.J.M.)

**Keywords:** anti-nicotine vaccine, cross-reactive material 197 (CRM_197_), smoking cessation, conjugation, Ab titer, Ab avidity

## Abstract

Smoking remains one of the major causes of morbidity and mortality worldwide. One approach to assisting smoking cessation is via anti-nicotine vaccines, composed of nicotine-like haptens conjugated to a carrier protein plus adjuvant(s). We have previously shown that the carrier, hapten, linker, hapten load, degree of conjugate aggregation, and presence of adducts can each influence the function (nicotine-binding capacity) of the antibody (Ab) induced. Herein, we extend those findings and show that tertiary structure is also critical to the induction of functional immune responses and that this can be influenced by conjugation conditions. We evaluated immunogenicity in mice using six lots of NIC7-CRM, a conjugate of 5-aminoethoxy-nicotine (Hapten 7), and a single point (glycine 52 to glutamic acid) mutant nontoxic form of diphtheria toxin, cross-reactive material 197 (CRM_197_), which were synthesized under different reaction conditions resulting in conjugates with equivalent molecular characteristics (hapten load, aggregates, adducts), but a different tertiary structure. When tested in mice, better functional responses (reduced nicotine in the brain of immunized animals relative to non-immunized controls) were obtained with conjugates with a more closed structure than those with an open conformation. These studies highlight the need for a better understanding of the physicochemical properties of small molecule conjugate vaccines.

## 1. Introduction

Smoking remains a significant cause of death and disease throughout the world contributing to the deaths of approximately 5.4 million people each year [1,2]. Although many options exist to help people who smoke quit such as nicotine replacement therapies (gum, patch) and prescription medicines (varenicline, bupropion), most people who smoke relapse and only 10–25% will remain abstinent at one year [3,4,5]. Anti-nicotine vaccines can provide another option via a novel mechanism of action. A vaccine targeting nicotine would promote the formation of antibody (Ab) to bind nicotine in the bloodstream before it can reach the brain. Preventing nicotine from reaching the brain prevents or minimizes the reward sensation associated with smoking, thus helping break the cycle of addiction [6].

At least five different nicotine vaccines have been tested in humans. Two of these (NicVax^®^, Nabi Pharmaceuticals (Rockville, MD, USA), and CYT002-NicQb, Cytos Biotechnology AG (Schlieren, Switzerland)) have been assessed in placebo-controlled Phase 2 studies. While neither study showed overall enhanced quit rates at one year compared to placebo, subgroup analyses in both studies showed improved quit rates in the top 30% of subjects for Ab titers, suggesting that the basic hypothesis was sound, but an insufficient number of subjects achieved the desired Ab response [7,8].

Nicotine by itself is not immunogenic and therefore cannot stimulate an Ab response; it must be attached to a larger immunogenic carrier molecule. We identified a nicotine-like hapten, Hapten 7, which, when conjugated to cross-reactive material 197 (CRM_197_), a non-toxic form of diphtheria toxin (DT), and injected into animals, was able to generate a robust immune response to nicotine [7]. We selected CRM_197_ since it has been successfully used as a carrier protein in a number of approved vaccines such as Prevnar^®^ (Pfizer, Clondalkin, Ireland) and Menveo^®^ (Novartis Vaccines and Diagnostics, Sovicille, Italy). Using this Hapten 7-CRM_197_ conjugate, we have demonstrated the key role that the hapten load, degree of conjugate aggregation, and presence of adducts, as well as the choice of adjuvant, play in determining Ab function in mice and non-human primates (NHP) [9,10,11]. Herein, we extend these studies and investigate the influence of the tertiary structure on the immunogenicity of the Hapten 7-CRM_197_ anti-nicotine vaccine, henceforth referred to as NIC7.

## 2. Materials and Methods

### 2.1. Preparation of NIC7 Conjugates Using Varied Concentrations of sNHS

A nicotine analog, (*S*)-2-(5-(1-methylpyrrolidin-2-yl)pyridine-3-yloxy)ethanamine mono-tosylate (Hapten 7) was synthesized as previously described [7]. The hapten was conjugated to cross-reactive material 197 (CRM_197_) (Pfizer, Sanford, NC, USA). Prior to conjugation, CRM_197_ was buffer exchanged using conjugation buffer (50 mM MOPS, 50 mM NaCl, pH 7.2). Protein concentration was determined by measuring A_280_ absorbance using an experimentally determined extinction coefficient of CRM_197_ of 0.94 (mg/mL)^−1^cm^−1^. A stock solution of 100 mg/mL Hapten 7 was made in conjugation buffer, and the pH was adjusted to pH 7.2. A stock solution of 50 mg/mL sulfo *N*-hydroxysuccinimide (sNHS) (Pierce, Rockford, IL, USA) was made in conjugation buffer and the pH was adjusted to pH 7.2. A stock solution of 100 mg/mL 1-ethyl-3-(3-dimethylamino)propyl carbodiimide hydrochloride (EDC) (Sigma-Aldrich, St. Louis, MO, USA) was made in conjugation buffer. The conjugation reactions were set up at a 30 mL total volume. Reaction components were added in the following order: CRM_197_, conjugation buffer, Hapten 7, sNHS. The mixture was then briefly mixed. EDC solution was added to initiate the reaction, and the reaction mixture was again mixed briefly. Each of the six reactions contained 0.000514 mmol of CRM_197_, 2.055 mmol of Hapten 7, and 1.9009 mmol of EDC. Only the sNHS concentration was varied for the six conjugation reactions. The varying levels of sNHS were 0.26, 0.51, 0.77, 1.34, 1.49, and 1.64 mmol to give sNHS/EDC ratios of 0.14, 0.27, 0.41, 0.7, 0.78, and 0.86, respectively. Reactions were incubated for 16 h at 16 °C. At the end of the incubation, each reaction was buffer exchanged into 10 mM histidine (Fisher Chemical, Fair Lawn, NJ, USA), 10 mM KPO_4_ (Fisher Chemical, Fair Lawn, NJ, USA), pH 7.0. Finally, an equal volume of 2× excipient buffer containing 10 mM histidine, 10 mM KPO_4_, 200 g/L sucrose (Fisher Chemical, Fair Lawn, NJ, USA), and 0.4 g/L PS80 (JT Baker, Phillipsburg, NJ, USA) was added to each conjugate. Conjugate protein concentration was determined by micro BCA (Thermo Scientific, Rockford, IL, USA).

### 2.2. Hapten Load by Acid Hydrolysis

A 200 µL sample was used for buffer exchange using 10,000 MWCO Amicon Ultra-4 filter (Millipore part of Merck KGaA, Darmstadt, Germany) and 10 mM potassium phosphate, 10 mM histidine, pH 7.0 to reduce the amount of sucrose. The protein concentration of the buffer-exchanged sample was determined using the BCA Protein Assay Kit (Sigma-Aldrich, St. Louis, MO, USA). One hundred micrograms of buffer-exchanged sample was transferred to a glass tube in a reaction vessel and acid-hydrolyzed for 90 min. The vessel was removed from the heated compartment and dried under vacuum and heat. Samples were reconstituted by pipetting 200 µL of 20 mM HCl into the tubes and vortexing to dissolve. Samples were analyzed using an Ace C18-AR column (4.6 × 150 mm, 3-µm particle, 100 Å pore size; Mac-Mod, Chadds Ford, PA, USA). A standard of Hapten 7 at 0.015 mg/mL (as free base) in 20 mM HCl was used for quantitation. The Hapten 7 peak was eluted with 0.2% trifluoroacetic acid (TFA) (Thermo Scientific, Rockford, IL, USA) in 98% H_2_O/2% acetonitrile (ACN) (Honeywell, Morristown, NJ, USA) within 8 min at a flow rate of 1 mL/min, column temperature of 40 °C, and UV detection at 280 nm. Injection volume was 10 µL. Hapten load was reported as mmol of Hapten 7 per mmol of CRM_197_. 

### 2.3. Size-Exclusion Chromatography (SE-HPLC) 

Samples were diluted to 0.25 mg/mL with 50 mM sodium phosphate, 500 mM sodium chloride, pH 6.0 and analyzed on a BioSuite 125 UHR SEC column (4.6 × 300 mm, 4 µm, Waters, Milford, MA, USA) using 50 mM sodium phosphate, 500 mM sodium chloride, pH 6.0, 2% acetonitrile as the eluent. Injection volumes were 50 µL. The flow rate was 0.3 mL/min isocratic at a column temperature of 30 °C for 35 min. Detection was at 214 nm.

### 2.4. Non-Reduced Capillary Gel Electrophoresis (CGE)

Samples consisted of a final volume of 110 µL at 1 mg/mL protein, 35 µL of proteome sample buffer (Beckman Coulter now AbSciex, Framingham, MA, USA), and the remaining volume of deionized water. Samples were vortexed, and 100 µL was then transferred to a micro vial (Applied Biosystems, Wilmington, DE, USA) and centrifuged for 3–5 min or until no bubbles were visible in the sample. Samples were analyzed using a ProteomeLab PA800 (Beckman Coulter now AbSciex, Framingham, MA, USA) equipped with an ultraviolet (UV) detector and Waters Empower chromatography data collection system (Milford, MA, USA). Samples were injected into a bare fused silica capillary 50 µm ID with an effective length of 10.0 cm and a total length of 30.2 cm with a 100 × 800 micron aperture. Separation occurred at 15 kV with pressure applied to both ends of the capillary at 20 psi. Detection was performed at 214 nm using a UV detector.

### 2.5. Matrix-Assisted Laser Desorption/Ionization Mass Spectrometry (MALDI-MS)

MALDI-TOF/TOF (Bruker Xtreme, Billerica, MA, USA) was used for MALDI analysis. Unconjugated CRM_197_ was used as a control. All samples were diluted to ~2 pmol/µL in 0.1% TFA in water. Two microliters of sample was diluted with 48 µL of 0.1% TFA. All samples were mixed 1:1 with a sinapinic acid MALDI matrix. The matrix was dissolved in 70% ACN containing 0.1% TFA at 20 mg/mL. Two microliters of sample with 2 µL of matrix were mixed together. One microliter of each sample was spotted onto a stainless steel MALDI target. The CRM_197_ control was spotted at each end of the sample rows to consider calibration drift across the plate. Samples were acquired in linear positive mode and calibrated with CRM_197_. A total of 10,000 shots were summed in 2000 shot increments.

### 2.6. Analytical Ultracentrifugation (AUC)

The test samples were diluted into formulation matrix consisting of 10 mM potassium phosphate, 10 mM histidine, 100 mg/mL sucrose, 0.2 mg/mL (Fisher Chemical, Fair Lawn, NJ, USA), and 0.2 mg/mL PS80 (JT Baker, Phillipsburg, NJ, USA) pH 7 (Fisher Chemical, Fair Lawn, NJ, USA) to obtain an absorbance reading of about 0.9 OD at 280 nm in the 1.2 cm centrifuge cell. The samples were analyzed using Beckman Analytical Ultracentrifuge, model XLI (Beckman-Coulter, Brea, CA, USA), spun at 40,000 rpm at 20 °C. The resulting data was analyzed using Sedfit (ver.11.8, National Institutes of Health, Bethesda, MD, USA) to generate c(s) size distribution plots and to determine sedimentation coefficients and the relative abundance of various species. 

### 2.7. Adjuvants

The B Class CpG of sequence 5′ TCG TCG TTT TTC GGT GCT TTT 3′ was synthesized with a nuclease-resistant phosphorothioate backbone (Avecia, Milford, MA, USA) as described previously [8]. Alhydrogel^®^ “85” (Brenntag Biosector, Frederikssund, Denmark) was the source of the aluminum hydroxide [Al(OH)_3_].

### 2.8. Animal Procedures

Female BALB/c mice (6–8 weeks old, Charles River Laboratories, Montreal, QC, Canada, *n* = 10/group) were immunized with 10 µg of each NIC7 conjugate in combination with Al(OH)_3_ (10 µg Al^3+^) and CpG (20 µg) made up to a total volume of 50 µL with PBS (Sigma-Aldrich, St. Louis, MO, USA). The vaccine formulations were administered by intramuscular (IM) injection in the left tibialis anterior (TA) muscle of mice lightly anaesthetized with Isoflurane^®^ (CDMV, St. Hyacinthe, QC, USA) on Days 0 and 21. Animals were bled on Days 20 and 27 by submandibular venus puncture using sodium heparin as an anti-coagulant, and recovered plasma was used for quantitation of nicotine-specific immune responses. In order to minimize any potential impact of bleeding on subsequent efficacy readouts, all animals had a similar volume of blood removed and were rehydrated by subcutaneous administration of Lactated Ringer’s solution (CDMV, St. Hyacinthe, QC, USA) to replenish blood volume. On Day 28, mice were challenged with ^3^H-nicotine, as described below. All groups were repeated at least once to ensure reproducibility of results. All procedures performed on animals in this study were in accordance with regulations and guidelines reviewed and approved by the Pfizer Institutional Animal Care and Use Committee and were conducted in facilities fully accredited by AAALAC International.

### 2.9. Anti-Nicotine Ab Titers

The levels of anti-nicotine IgG Ab in mouse plasma were quantified by endpoint ELISA (in duplicate) for individual animals using 96-well plates coated with a nicotine derivative, rac-trans-3’-thio methyl nicotine dihydrochloride, (Toronto Research Chemicals, North York, ON, Canada) conjugated to bovine serum albumin (Sigma-Aldrich, St. Louis, MO, USA) as previously described [9]. Titers were defined as the highest plasma dilution that resulted in an absorbance value (OD 450) twofold greater than that of non-immune plasma, with a cut-off value of 0.05. Anti-nicotine Ab levels are shown as a geometric mean titer (GMT) +/− 95% confidence interval (CI). 

### 2.10. In Vivo Functional Assays 

One week after the second immunization, animals were individually weighed such that each mouse received a tail vein infusion of 0.05 mg/kg of nicotine hydrogen tartrate (Sigma-Aldrich, St. Louis, MO, USA) containing 3 μCi ^3^H-nicotine (Perkin Elmer, Waltham, MA, USA) in 100 μL of PBS; at 5 min post-infusion, the animal was then bled and perfused, after which the brain was removed and weighed. The levels of ^3^H-nicotine per mg of brain or mL of blood were measured by liquid scintillation counting, and the percent change relative to control non-immunized mice was calculated. Ab function is shown as % reduction of nicotine in brain (+/− SD) or % increase of nicotine in plasma (+/− SD) compared to control non-immunized mice. Details of these procedures have been described previously [9]. The 0.05 mg/kg dose of nicotine used is considered approximately equivalent to the mg/kg dose of nicotine in humans from three smoked cigarettes [12]. 

### 2.11. Statistical Analysis 

In vivo data were analyzed using GraphPad Prism (GraphPad Software, San Diego, CA, USA). Statistical significance between groups of mice was calculated by 1-factor analysis of variance (ANOVA) followed by post-hoc analysis using either Dunnett’s (comparison with control group) or Tukey’s (comparison between groups) multiple comparison tests. Differences were considered to be non-significant with *p* > 0.05.

## 3. Results

### 3.1. Effect of Varying sNHS/EDC Ratios on Main 1, Main 2, and Hapten Load

**Figure 1:**
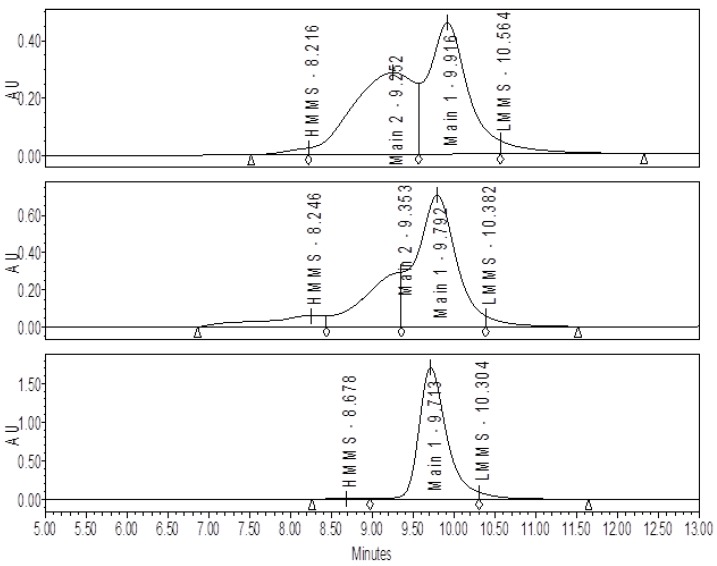
Size-exclusion chromatography (SE-HPLC) chromatograms for NIC7 conjugates made with high and low ratios of sulfo *N*-hydroxysuccinimide (sNHS) and 1-ethyl-3-(3-dimethylamino)propyl carbodiimide hydrochloride (EDC). The top panel shows the SE-HPLC profile for a conjugate produced with a sNHS/EDC ratio of 0.14. For this sample, the amount of Main 1 vs. Main 2 is almost 1:1. The middle panel shows the SE-HPLC profile for a conjugate produced with a sNHS/EDC ratio of 0.86 and Main 2 is present to a much lower extent. The bottom panel shows a control sample of unconjugated CRM_197_ showing that Main 2 is not present.

**Figure 2:**
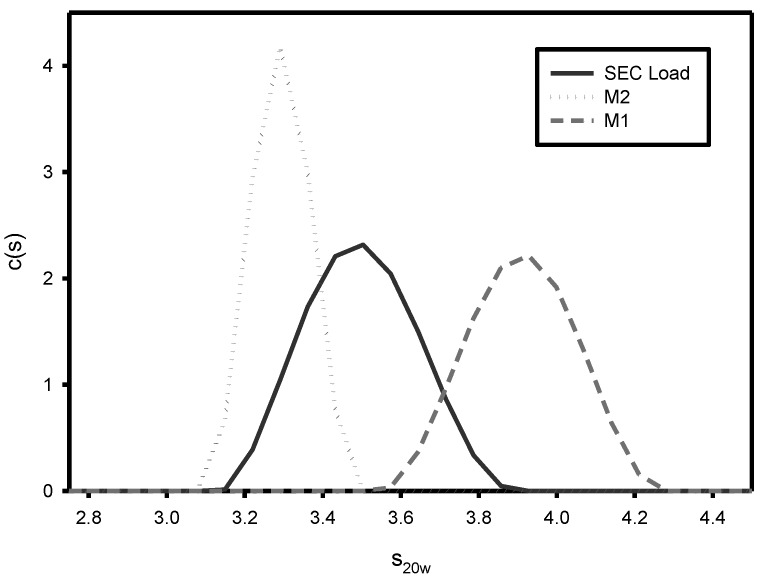
Analytical ultracentrifugation (AUC) analysis of Main 1 vs. Main 2. Fractions from SE chromatography were collected and analyzed by AUC. Fractions corresponding to Main 1 (dashed line) and Main 2 (dotted line) show significantly different sedimentation coefficients. The black trace is from a sample of the load material for the SE-HPLC run.

A comparison by SE-HPLC analysis of conjugates produced with a high level (0.86) versus a low level (0.14) of sNHS/EDC shows the presence of one major peak (Main 1) with a shoulder peak (Main 2) present on the faster eluting side of Main 1 (Figure 1). At the higher sNHS/EDC ratio (0.86), Main 2 comprises about 25% of the major peak, while at the lower ratio (0.14) it increases significantly to 47% of the major peak. The presence and size of Main 2 is directly attributable to the conjugation reactions conditions since this peak does not appear in samples of purified, unconjugated CRM_197_ (Figure 1). Analytical ultracentrifugation (AUC) (Figure 2) and SEC-MALLS (data not shown) indicate that Main 1 and Main 2 are both monomeric NIC7 conjugates; however, the slower sedimentation coefficient for Main 2 from the AUC data suggests that Main 2 has a more open conformation than Main 1.

**Figure 3:**
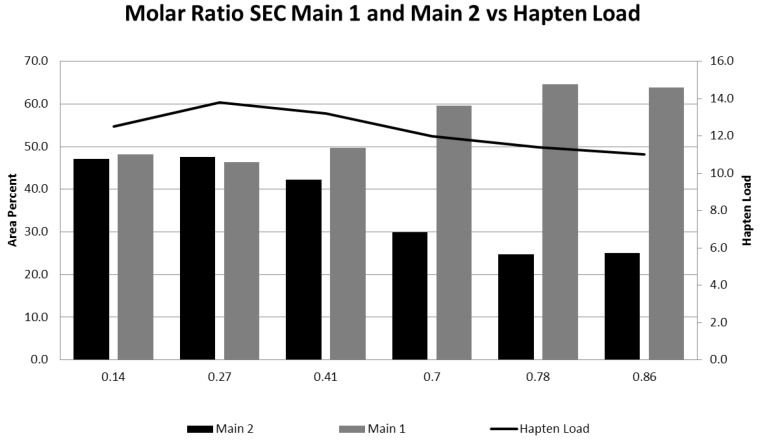
The effect of varying sNHS/EDC ratios on Main 1, Main 2, and hapten load. The amounts of Main 1 and Main 2 for conjugates at each ratio were determined by integrating the area under the peaks from SE-HPLC. Main 1 is represented by the light gray columns, and Main 2 by the dark gray columns. The hapten load for each sample as determined by acid hydrolysis is shown by the curve.

As the sNHS/EDC molar ratio is decreased from a high of 0.86 to a low of 0.14, the amount of Main 2 increases indicative of a more open conformation; however, the hapten load, which is an overall average, and levels of high molecular mass species (HMMS) as measured by CGE remain fairly constant (see Table 1, Figure 3).

### 3.2. The Effect of Different sNHS/EDC Ratios on Anti-Nicotine Responses

**Figure 4:**
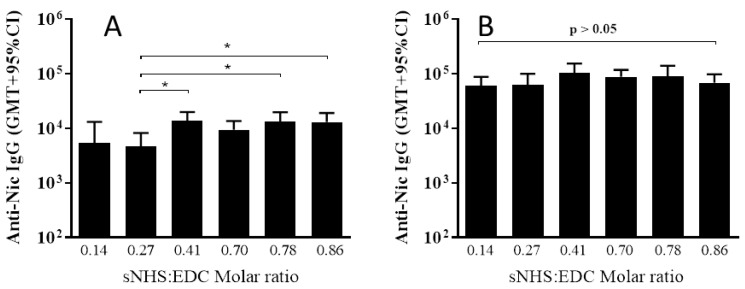
Effect of different sNHS/EDC ratios on antibody (Ab) titers. BALB/c mice (*n* = 10/group) were immunized on Days 0 and 21 by IM injection with 10 µg of different NIC7-CRM conjugates adjuvanted with alum (10 µg Al^3+^) + CpG (20 µg). Plasma was collected on Day 20 and 28 and anti-nicotine antibody levels determined by ELISA. Anti-nicotine Ab titers post-Dose 1 and 2 are shown in Panels (**A**) and (**B**), respectively. Statistical significance was calculated by a one-factor analysis of variance (ANOVA) followed by Tukey’s post-hoc multiple comparison test. * *p* < 0.05.

All six adjuvanted NIC7 conjugates generated nicotine-specific Ab in mice (Figure 4). Following a single administration to mice, there were some differences in Ab titers (*p* = 0.018; one-way ANOVA) between conjugates made using lower sNHS/EDC molar ratios and those that were made at higher ratios, but differences were small (2- to 3-fold difference in geometric mean titer (GMT)), and boosting all conjugates induced equivalent titers (*p* = 0.146; one-way ANOVA).

**Figure 5:**
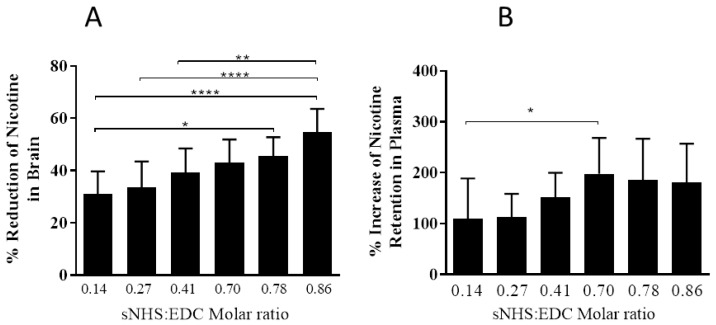
The effect of different sNHS/EDC ratios on Ab function. BALB/c mice (*n* = 10/group) were immunized on Days 0 and 21 by IM injection with 10 µg of NIC7-CRM conjugates adjuvanted with alum (10 µg Al^3+^) + CpG (20 µg). On Day 28, animals received an IV injection of ^3^H-nicotine (0.05 mg/kg) and plasma and brains collected. Panel (**A**) shows % reduction of nicotine levels in brain (ng-eq/g), and Panel (**B**) shows % increase of nicotine levels in plasma (ng-eq/mL) compared to control non-immunized mice. * *p* < 0.05; ** *p* < 0.01; **** *p* < 0.

While all six NIC7 conjugates significantly increased nicotine in the plasma (*p* < 0.0001; 1-way ANOVA followed by Dunnett’s post-hoc multiple comparison test compared to control mice) and significantly reduced nicotine in the brain compared to control non-immunized mice (*p* < 0.0001; 1-way ANOVA followed by Dunnett’s post-hoc multiple comparison test compared to control mice), better functional responses were obtained with NIC7 conjugates made at an sNHS/EDC ratio of ≥0.7 and weaker responses with conjugates made at ratios below this (Figure 5).

## 4. Discussion

Biophysical characterization of NIC7 conjugates under varying conjugation reaction conditions tested indicates that two major conformations exist in solution: Main 1, which is a more “closed” conformation according to AUC data, and Main 2, which has a more “open” conformation. The presence of these two conformations for a CRM_197_-based conjugate where one conformation is more “open” than the other is not surprising given the evidence for such conformations for DT, the parent molecule of CRM_197_. Multiple studies indicate that the pH, temperature, buffers, and other factors can affect the stability of DT and lead to a partial unfolding of the molecule while maintaining a defined tertiary structure [13,14]. 

The existence of a partially unfolded form of DT is also supported by the comparison of the crystal structures of monomeric and domain-swapped DT. Under certain conditions, DT can form a dimer by swapping the R domain between monomers, which requires an almost 180° movement of the R domain [15]. Given that the unfolding of DT is necessary for the molecule to enter cells through the cell membrane as part of its normal pathogenesis, the ability of DT to unfold is actually a necessary property of the molecule. Since the crystal structures of DT and CRM_197_ are nearly superimposable, with the only difference being increased flexibility of the active-site loop in front of the NAD binding pocket, the assumption being that CRM_197_ can undergo a similar “open” vs. “closed” transformation [16].

The conformational change observed for the conjugate molecules seems to be driven by the conjugation reaction conditions, specifically the level of sNHS in the reaction. Keeping all other variables (except for the sNHS level) constant results in conjugates that differ only in their SE-HPLC profiles, which is indicative of the molecular conformation. Other key factors such as hapten load and HMMS levels remained essentially the same regardless of the sNHS level. 

The presence of sNHS increases the efficiency of the conjugation reaction. Conjugation with EDC alone produces an unstable o-acylisourea intermediate. The addition of sNHS converts this unstable intermediate into a more stable sNHS ester intermediate increasing the efficiency of the conjugation reaction [17]. Therefore, as sNHS levels increase, so too should the level of conjugation. Since conjugation can occur not only between separate CRM_197_ molecules, but between any free amino and carboxyl groups, the number of intra-protein cross-links should also increase. Intra-protein cross-linking can change the stability and structure of a protein [18,19] and therefore could be the driving force causing the transition from the “closed” to the “open” conformation.

Perhaps surprisingly, the increase in conjugation predicted by higher sNHS levels does not result in higher hapten load. This is most likely due to competing side reactions and the overall low efficiency of the conjugation of hapten to CRM_197_. Previous studies investigating the kinetics of EDC/sNHS conjugation using a model amine and model peptides indicate several competing side reactions. These side reactions include interactions among EDC, sNHS, and the amine, intra-peptide conjugation, and hydrolysis of EDC and sNHS all of which compete for conjugation of the amine to the peptides [20]. By analogy, we would expect the same competing reactions and similar conjugation efficiencies for the conjugation of Hapten 7 to CRM_197_ and would also expect increased levels of sNHS to have a minimal impact on hapten load; however, the role of sNHS in promoting a conformational change in CRM_197_ requires further study to determine the exact mechanism of action.

Although both Main 1 and Main 2 are monomeric NIC7 conjugates, they have different conformations with Main 2, which possesses a more unfolded or “open” configuration compared to Main 1. While this does not seem to impact their ability to induce nicotine-specific Ab, it does appear to influence the quality or functionality of the induced Ab. For example, with conjugates obtained using lower sNHS/EDC ratios, corresponding to a higher percentage of Main 2 and therefore more open conformation, the anti-nicotine Ab function decreased (as evidenced by a decreased ability to sequester nicotine in the blood and prevent its entry into the brain). It is well-established that the Ab function is determined both by the Ab level and avidity, and it is possible that the loss of Ab function with Main 2 observed in our studies may have been due to changes in Ab avidity. The loss of avidity could be due to a change in the way the hapten groups are presented to the B cells in the “open” vs. “closed” conformation, resulting in B cell epitopes that are lost or conform suboptimally to B cell receptors. Since antigen-specific Ab levels were not affected, yet function was, it is also possible that T helper epitopes on CRM_197_ may also have been altered as a result of the conformational change. 

Unfolding of CRM_197_ upon conjugation has been previously reported with other antigens [20] and conformational changes to either carrier or antigen have been shown to impact the efficacy of a number of other vaccines, highlighting the importance of physicochemical characterization [21,22,23,24].

## 5. Conclusions

Overall, the results of this study demonstrate the need for a better understanding of the physicochemical properties of small molecule conjugate vaccines. We have previously shown that the selection of the nicotine-derived hapten, carrier, conjugation chemistry, hapten load, molecular size (aggregation), presence of adducts, and adjuvant selection can all impact the immunogenicity of anti-nicotine vaccines. We here show that tertiary structure is also critical to the induction of functional immune responses. This may help lead to the development of more effective vaccines that result in better clinical outcomes.

## Figures and Tables

**Table 1:** % Main 1, Main 2, high molecular mass species (HMMS), and hapten load for samples with varying sNHS/EDC ratios. CGE, Capillary Gel Electrophoresis.

Molar Ratio sNHS:EDC	SE-HPLC		CGE %HMMS	Hapten Load
	%Main 2	%Main 1		
0.14	47.1	48.2	2.3	13
0.27	47.6	46.4	2.3	14
0.41	42.3	49.6	3.0	13
0.70	29.9	59.6	1.8	12
0.78	24.8	64.6	2.0	11
0.86	25.0	63.9	2.6	11
